# Beyond Hormones: A Systematic Review of the Risk of Cardiovascular Diseases in Polycystic Ovary Syndrome

**DOI:** 10.7759/cureus.72987

**Published:** 2024-11-04

**Authors:** Chandrani Dutta, Srivarshini Maddukuri

**Affiliations:** 1 Family Medicine, Dr. D. Y. Patil Medical College, Hospital and Research Centre, Pune, IND; 2 Internal Medicine, Dr. D. Y. Patil Medical College, Hospital and Research Centre, Pune, IND

**Keywords:** atherosclerotic cardiovascular disease, endocrine disorders, inflammatory biomarkers, medical screening, metabolic syndrome (mets), polycystic ovary syndrome (pcos), prevention of cardiovascular diseases, public health awareness, risk factors of cardiovascular diseases, stein-leventhal syndrome

## Abstract

Polycystic ovary syndrome (PCOS) is a common endocrinopathy among women in the reproductive age group. PCOS is defined by the Rotterdam criteria, which include hyperandrogenism, oligo-anovulation, and polycystic ovaries on ultrasound. The common symptoms are irregular or absent periods, acne, hirsutism, and alopecia androgenica. Increased prevalence of metabolic syndrome is seen among women with PCOS, which increases cardiovascular risk (CVR). Insulin resistance (IR), being most common in PCOS, is often seen in patients with obesity. IR and obesity induce chronic low-grade inflammation in PCOS, increasing various inflammatory markers. Increase in the parameters of tumor necrosis factor-alpha (TNF-alpha), interleukin-1 (IL-1), interleukin-6 (IL-6), C- reactive protein (CRP), plasminogen, endothelin-1, fibrinogen, cystatin-C, fetuin B, vascular endothelial growth factor, and endostatin levels have been documented in the PCOS-affected women. Microbiota alteration is also seen in this demographic, which increases metabolites like trimethylamine-N-oxide (TMAO). TMAO, because of its pro-atherosclerotic activity, is linked to an increase in CVR. In this systematic review, we intended to discover the causes and factors that lead to increased CVR in women diagnosed with PCOS. This systematic review used PubMed, regular keywords, and Google Scholar. The inclusion criteria included the human female population of all ages and literature available in the English language in free full text published between the years 2019 and 2024. The exclusion criteria included research involving animals, works published before 2019, articles written in a language other than English, and articles not publicly available. A total of 89 articles were identified, and 8 final articles were selected after quality assessment.

## Introduction and background

The Rotterdam criteria define polycystic ovary syndrome (PCOS) as oligo-ovulation or chronic anovulation, clinical or biochemical hyperandrogenism, and polycystic appearance of ovaries on ultrasound [1]. According to data, it is a complex metabolic and reproductive disease that affects between 5% and 18% of women of the reproductive age group [2,3]. Up to 40-80% of those women are overweight or obese [4]. Many metabolic diseases have been linked to it, such as insulin resistance (IR), obesity, hypertension, dyslipidemia, hyperandrogenism, type 2 diabetes mellitus (T2DM), and chronic low-grade inflammation, which eventually increase the cardiovascular risk (CVR) [2,3,5,6].

Cardiovascular diseases (CVDs) are also the leading cause of death among women, contributing up to 35% of deaths in women each year. It is mentioned in several studies that women with PCOS have an inflated risk of developing CVDs [2,3,6,7,8,9]. The increase in cardiometabolic diseases in association with this syndrome warrants screening for CVR to prevent severe consequences [10].

IR, which is a strong predictor of atherosclerotic CVD and consequent hyperinsulinemia, is a common finding recorded in this disease population, and one of the most common causes of IR is obesity and overweight [2,9,11]. However, IR is also proven to be independent of weight in women with PCOS; therefore, lean patients can also display hyperinsulinemia, ultimately leading to T2DM [9,10]. The increased risk of CVD in PCOS [12], is also linked to hyperandrogenism, which is a trademark finding of PCOS, and has a clear association with an increased prevalence of cardiometabolic disorders [5,13].

In addition to the metabolic diseases associated with PCOS [14], a disturbance in the oxidant-antioxidant balance in patients with polycystic ovaries increases oxidative stress [6]. Chronic low-grade inflammation commonly encountered in PCOS-affected subjects is a contributing factor in the development of IR and accelerated atherogenesis as it creates a pro-inflammatory environment in the body [2,6,7]. Several inflammatory markers and mediators have been studied and associated with this syndrome [6], which are discussed in detail in this systematic review.

There are characteristic increased levels of serum triglycerides, total cholesterol, and decreased high-density lipoprotein cholesterol (HDL-C), although this is not a diagnostic criterion for PCOS, it contributes to adverse cardiovascular effects [2,11,13,15]. This systematic review has discussed in detail the role of obesity, IR, hyperandrogenism, dyslipidemia, various markers and metabolites of chronic low-grade inflammation, genetics, and biochemical, physiological, and imaging changes that suggest CVR enhancement in women with PCOS. This review aims to understand the proposed pathophysiology of PCOS, identify the risk factors that promote adverse cardiac outcomes in women with PCOS, and evaluate the need for screening in the affected population.

## Review

Methods

Our systematic review was conducted following the Preferred Reporting Items for Systematic Reviews and Meta-Analyses (PRISMA) 2020 statement. We used advanced search on Google Scholar and PubMed with regular and MeSH keywords. Inclusion criteria included a human female population of reproductive age group, available in English language and free full text published between 2019 and 2024. The exclusion criteria included research involving animals and pre-menarche and menopausal women, works published before 2019, articles written in languages other than English, and works not publicly available.

A PubMed search found 32 articles, whereas an advanced search on Google Scholar generated 153 articles. Relevant papers are selected after the inclusion and exclusion criteria shown in Table 1.

**Table 1:** Databases, search strategy and keywords, filters, and results generated and used in this systematic review

Database name	Search strategy and keywords	Filters applied	Results
PubMed	(“Polycystic Ovary Syndrome/complications” OR “Polycystic Ovary Syndrome/diagnosis” OR ”Polycystic Ovary Syndrome/epidemiology” OR “Polycystic Ovary Syndrome/mortality” OR “Polycystic Ovary Syndrome/prevention and control” OR “Polycystic Ovary Syndrome/therapy”) AND “Risk Factors”) AND ( “Cardiovascular Diseases/complications” OR “Cardiovascular Diseases/diagnosis” OR “Cardiovascular Diseases/drug therapy” OR “Cardiovascular Diseases/epidemiology” OR “Cardiovascular Diseases/etiology” OR “Cardiovascular Diseases/prevention and control”)	Human female population of all ages Languages: English, free full text available between 2019 and 2024	32 articles
Google Scholar	“polycystic ovary syndrome and cardiovascular disease; cardiovascular risk in women with PCOS”	An advanced search was done.	153 articles

Results

Study Selection and Quality Assessment

We included all relevant studies assessing CVR in women with PCOS based on predefined eligibility criteria. All references were entered into EndNote. The initial selection was done based on the study titles, followed by deleting duplicates and reviewing the abstracts of all remaining records. A total of 89 articles were obtained after applying the inclusion/exclusion criteria and duplicate removal. Full-text articles were obtained for review and data processing for all selected abstracts. After reviewing full-text articles, 28 articles were selected for quality appraisal by two authors individually.

Quality appraisal was done using the Newcastle-Ottawa Scale (NOS), Scale for the Assessment of Narrative Review Articles (SANRA), Assessment of Multiple Systematic Reviews (AMSTAR), Joanna-Briggs Institute (JBI), and Cochrane Risk of Bias tool 2.0. Each assessment tool had its own criteria and scores for quality review, and 8 articles were selected with scores above 70%.

**Figure 1:**
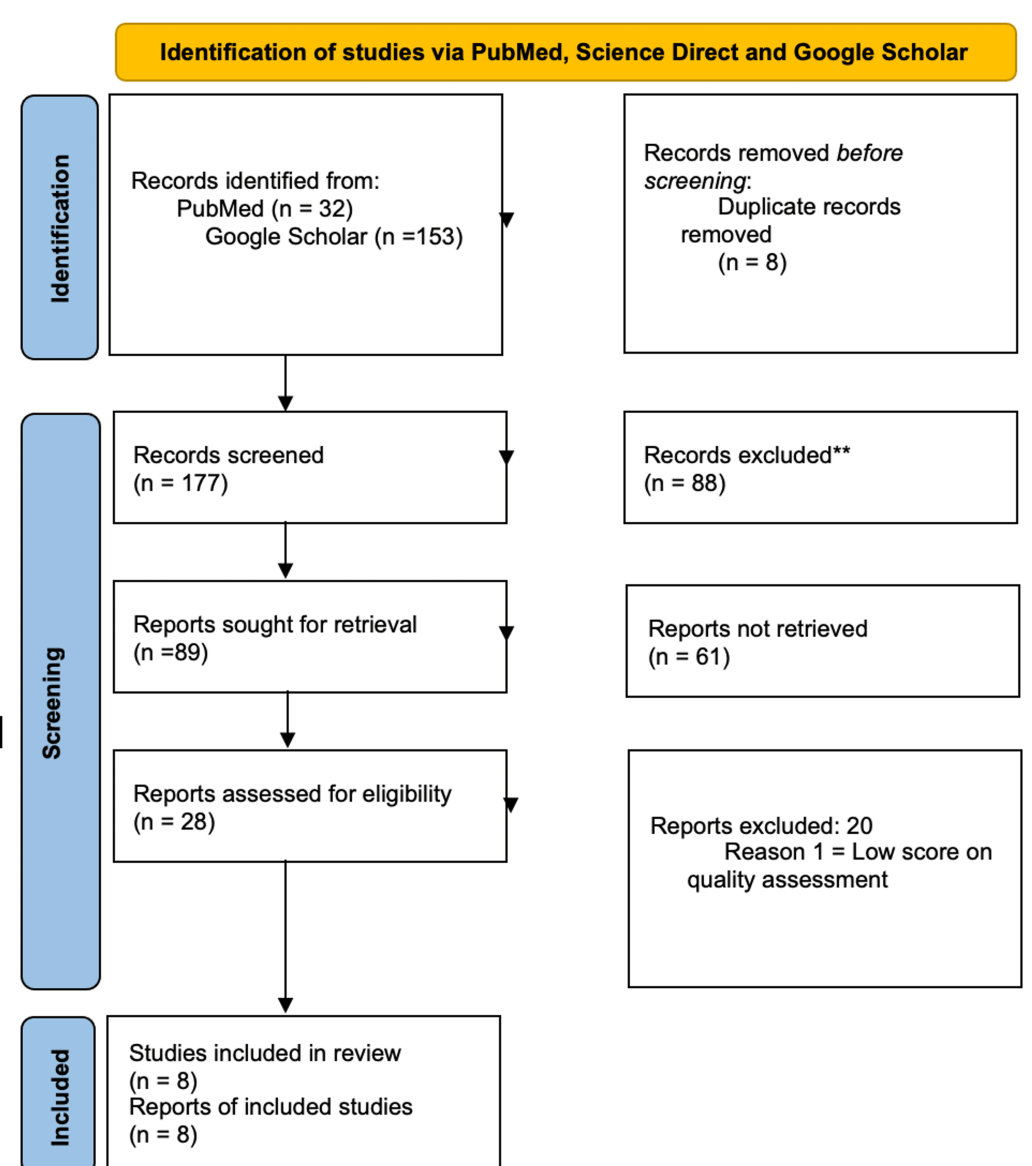
PRISMA 2020 flow diagram for new systematic reviews, which included searches of databases and registers only PRISMA: Preferred Reporting Items for Systematic Reviews and Meta-Analyses [16]

The Preferred Reporting Items for Systematic Reviews and Meta-Analyses (PRISMA) flow diagram for search databases is shown in Figure 1, and tools used for quality assessment of studies are shown in Table 2.

**Table 2:** Quality Assessment tools and scores of each study selected for this systematic review SANRA: Scale for the Assessment of Narrative Review Articles; NOS: Newcastle-Ottawa Scale; AMSTAR: Assessment of Multiple Systematic Reviews; JBI: Joanna Briggs Institute

Study no.	Study name	Type of study	Quality assessment tool used	Score (acceptable score > 70%)
1.	Donma O et al. [1]	Case control	NOS	77%
2.	González F et al. [2]	Case control	NOS	87.5%
3.	Bayındır A et al. [3]	Cross-sectional study	NOS	87.5%
4.	Jafari S et al. [4]	Quasi-experimental study	JBI	88%
5.	Amiri M et al. [5]	Systematic review, meta-analysis, and meta regression	AMSTAR	75%
6.	Başer Ö Ö et al. [6]	Case control	NOS	87.5%
7.	Falcetta P et al. [7]	Cross-sectional	NOS	100%
8.	Stuenkel C. A et al. [8]	Not specified	SANRA	75%

Study Characteristics

The study characteristics of each selected article are summarized and shown in Table 3.

**Table 3:** Main study characteristics of the selected studies accepted in this review PCOS: polycystic ovary syndrome; LDL: low-density lipoprotein; HLD: high-density lipoprotein; TNF: tumor necrosis factor; CRP: C-reactive protein; CVD: cardiovascular disease

Study no.	First author and year	Study type	Disease	Inclusion and exclusion criteria	Sample size/no. of studies	Outcome and key points
1.	Donma O et al., 2019 [1]	Case control study	Interpretation of molecular linkages between inflammation and angiogenesis in PCOS etiology	Inclusion criteria: 33 women with PCOS diagnosed as per the Rotterdam criteria and 31 controls without PCOS and devoid of chronic diseases, aged between 18 and 39 years, were included in the study.	64	This study found a statistically significant increase in endostatin levels in PCOS. Endostatin inhibits angiogenesis (formation of new vessels) and lowers vascular endothelial growth factor (VEGF). Endothelin is also a cardiovascular biomarker. The cardiometabolic risk factors including endothelial dysfunction is seen in PCOS women. The rise in endothelin level thereby signifies vascular and myocardial damage in PCOS women.
2.	González F et al., 2021 [2]	Controlled clinical trial	Saturated fat ingestion stimulates proatherogenic inflammation in PCOS	Inclusion criteria: Study subjects: 20 women with PCOS consisting of 10 lean and 10 obese subjects of 18-35 years of age exhibiting polycystic ovaries on ultrasound. None of the subjects used medications or smoked tobacco that could interfere with the outcome of the study. Controls: 20 controls consisting of 10 lean and 10 obese subjects of 19-40 years of age with regular menses, normal androgen levels, and no sign of polycystic ovaries without PCOS but with similar BMI as study subjects were included in the study. Exclusion criteria: Non-classic congenital adrenal hyperplasia, Cushing syndrome, hyperprolactinemia, thyroid disease, diabetes and inflammatory illnesses were excluded in all subjects.	40	In PCOS, ingestion of saturated fat stimulates proatherogenic inflammation independent of obesity. However, the combination of PCOS and obesity causes a more significant inflammatory response than obesity alone. Pro-inflammatory marker, heat shock protein-70 was significantly higher in PCOS women with obesity as compared to control subjects with obesity alone. Parameters like plasma cholesterol, LDL, and triglycerides were higher in PCOS women while plasma HDL was lower as compared to controls. Lean women with PCOS have greater amount of abdominal fat and are also at a greater risk of developing cardiovascular diseases as compared to lean control subjects.
3.	Bayındır A et al., 2020 [3]	Cross-sectional study	Fetuin B levels in PCOS and its association with carotid intima media thickness	Inclusion criteria: Subjects: 80 women diagnosed with PCOS as per the 2003 Rotterdam consensus criteria who fulfilled all three criteria for 1) oligo and/or anovulation, 2) clinical and/or biochemical signs of hyperandrogenism, and 3) ultrasonographic findings of polycystic ovaries. Control: 80 healthy women with regular menstrual cycles and no connected health problems were BMI and age matched without PCOS were included. Exclusion criteria: Other causes of irregular menstrual cycle and/or androgen excess like hyperprolactinemia, cushing’s syndrome, diseases of thyroid and adrenal glands, galactorrhoea, breastfeeding and pregnancy, impaired glucose tolerance, diabetes mellitus type 2, hypertension, hyperlipidemia, congestive heart failure, liver or renal failure, coronary artery disease, acute infections, known malignancy, inflammatory or autoimmune diseases, use of hormonal contraception and/or anti-androgen therapy, BMI <18.5 kg/m^2^ or >35 kg/m^2^, age <18 or >35 and medications that could affect the outcome of this study.	160	Fetuin-B levels and carotid intima media thickness (CIMT) were assessed in PCOS women. Circulating fetuin-B levels were significantly elevated in PCOS patients with IR. Increased fetuin-B levels is closely linked to hormonal-metabolic disturbance and increases cardiovascular risk in women with PCOS. CIMT, which is a good indicator of cardiovascular risk, was also found to be increased in PCOS subjects as compared to controls.
4.	Jafari S et al.. 2020 [4]	Quasi-experimental study	PCOS	Inclusion criteria: Obese women with PCOS between the ages of 20-30 years with no history of cardiovascular, renal or liver diseases, diabetes, no use of hormonal contraceptives and no smoking or alcohol intake were included in the study.	24	Aerobic exercise is beneficial in the treatment of PCOS and should be advised as a technique to manage PCOS to reduce the risk of cardiovascular adversity. In this study, BMI, insulin resistance, and inflammatory markers like TNF-alpha and CRP reduced as a result of long-term aerobic exercise in women with PCOS.
5.	Amiri M et al., 2020 [5]	Systematic review, meta-analysis, and meta regression	Hypertension in PCOS	Hypertension in women with PCOS	30 studies	PCOS women exhibit a higher risk of hypertension when compared to controls; however, this difference does not extend to menopausal women who had PCOS in the past. This could be due to the decreasing levels of androgen levels in blood as age progresses leading to reduction in cardiovascular risk factors.
6.	Başer Ö Ö et al., 2022 [6]	Case control study	Cystatin-C levels, Inflammatory, oxidant and antioxidant markers in PCOS	Inclusion criteria: Patients over the age of 18 years between Jan 1, 2022 and Apr 1, 2022 were included in the study. Exclusion criteria: Chronic systemic disease, infectious and inflammatory disease, psychiatric disorder and it’s treatment, thyroid dysfunction, history of bariatric surgery, congenital adrenal hyperplasia, androgen-secreting tumours, Cushing’s syndrome, hyperprolactinoma and adrenal disorders, hormone replacement therapy, or use of oral contraceptives or drugs for insulin resistance and less than 18 years of age were excluded.	96	Cystatin C is a predictor of all-cause mortality, such as cardiovascular disease and diabetes mellitus. This study found high levels of cystatin C in PCOS patients and high levels of inflammatory markers like interleukin-1 (IL-1), interleukin-6 (IL-6), tumor necrosis factor alpha (TNF-alpha), and malinaldehyde (MDA) and low levels of antioxidant like superoxide dismutase (SOD) in PCOS women. These findings indicate a need for addition of antioxidant and anti-inflammatory agents for cardiovascular protection in PCOS women.
7.	Falcetta P et al., 2021 [7]	Cross-sectional study	Clinical features, effect of aging, and metabolic abnormalities of PCOS women	Inclusion criteria: Women diagnosed with PCOS using the International PCOS network guidelines between February 1, 2014 and November 30, 2019; PCOS women with clinical or biochemical evidence of hyperandrogenism with oligo-amenorrhea for at least two years; PCOS women with gynecological age >2 years; were included. Exclusion criteria: Patients with idiopathic hyperandrogenism, idiopathic hirsutism, ovarian or adrenal tumor diagnosis, and hyperprolactinemia were excluded.	602	Ovarian dysfunction and hyperandrogenism, expressed as free androgen index (FAI), are key concerns in young PCOS women, and metabolic burden increases with age. However, metabolic abnormalities are commonly seen in PCOS women regardless of age. Authors found that higher BMI plays an important role in the metabolic sequelae in PCOS independent of age. Hyperandrogenism and weight gain must be prevented and managed early to prevent metabolic abnormalities in PCOS women.
8.	Stuenkel C A et al., 2024 [8]	White paper	Cardiovascular risk in women with PCOS and reproductive milestones	-	-	A growing number of reproductive milestones have been linked to increased risk of cardiovascular disease (CVD) in women. Early implementation of preventative strategies, when the reproductive milestone is first identified, is expected to enhance CVD outcomes. Women with PCOS should be considered at an increased risk for cardiovascular diseases and screening should be implemented.

Discussion

Rotterdam criteria is the most widely and commonly used diagnostic criteria for PCOS. Another commonly used criterion is by the National Institute of Health (NIH) [14,17].

The Rotterdam criteria define PCOS as oligo-ovulation or chronic anovulation, clinical or biochemical hyperandrogenism (HA), and polycystic appearance of ovaries on ultrasound [1,9,18].

Phenotypes

There are four established phenotypes of PCOS, according to the NIH [14,19]: 1) Phenotype A: HA, ovulatory dysfunction (OD), and polycystic changes (PCO). 2) Phenotype B: HA and OD. 3) Phenotype C: HA and polycystic changes (PCO). 4) Phenotype D: non-hyperandrogenic PCOS, i.e., OD and PCO.

Phenotypes A (HA + OD + PCO) and B (HA + OD) are termed classical PCOS, and phenotypes C (HA + PCO) and D (OD +PCO) are termed non-classical PCOS. The prevalence of phenotype A is 67.7%, which makes it the commonest phenotype, followed by phenotypes B, C, and D, with a prevalence of 11%, 17.7%, and 3.6%, respectively [20]. Phenotype D is the only non-hyperandrogenic phenotype with less risk of obesity, insulin resistance (IR), abnormal lipid profile, and metabolic changes [20]. However, despite different phenotypes, CVR factors in women with PCOS are increased in all phenotypes [1].

Clinical Presentation

Signs and symptoms seen in PCOS are due to hormonal changes. Symptoms of HA include acne, hirsutism, and alopecia androgenica [9,10]. Reproductive symptoms like infertility, anovulation, polycystic ovaries, psychological symptoms like anxiety-depressive symptoms, eating disorders, and body image issues also co-exist [11,12,17]. There is also an increased prevalence of dyslipidemia, T2DM, metabolic syndrome, obesity, hypertension, IR, endothelial dysfunction, chronic low-grade inflammation, and HA, which augment the risk for atherosclerotic CVD [15]. It has also been reported that the affected women are diagnosed in adolescence; however, many women with the syndrome are undiagnosed until they seek treatment for infertility [17].

Epidemiology

According to data, PCOS is a complex metabolic and reproductive disease that affects between 5% and 18% of women of the reproductive age group [2,3]. Up to 40-80% of women with PCOS are overweight or obese [4,11]. The prevalence of metabolic syndrome in women with PCOS ranges from 43% to 46% [21].

Exploring the Mechanisms of CVR in Women Diagnosed With PCOS

PCOS is a multifactorial disease with unknown etiology and pathophysiology [9,12]. Still, many metabolic diseases have been linked to the disease, such as IR, obesity, hypertension, dyslipidemia, HA, T2DM, and chronic low-grade inflammation, which eventually increase the CVR [2,3,5,6,9,13,14,15,22]. There is evidence of increased prevalence of CVDs like subclinical atherosclerosis, endothelial dysfunction, increased carotid intima thickness (CIMT), and coronary artery calcification, which is two- to four-fold higher in patients with PCOS compared to the general population [5,23].

Several studies have shown that women suffering from PCOS display higher arterial blood pressure (BP) readings as compared to healthy women [10,11]. However, the BP is only mildly elevated and does not warrant treatment as per clinical guidelines [24]. In women with this syndrome, increased readings of BP are independent of obesity or BMI, but it is exacerbated by obesity [14,24].

It has been mentioned in several studies that the risk of developing CVDs is higher among women diagnosed with PCOS [2,3,6,7,9,12,15,18,22]. CVD is also the leading cause of death among women, contributing up to 35% of deaths in women each year [8,11].

In a narrative review by Guan et al. [14], the authors found numerous articles containing cohort studies, retrospective studies, and meta-analyses that showed a higher prevalence of CVDs among women suffering from PCOS than among controls. Alternatively, the review also found studies that did not find any significant association of increased CVR in premenopausal or postmenopausal women with PCOS as compared to controls. The authors stated that the conflicting findings may be due to different diagnostic criteria, unavailability of specific biomarkers, the inclusion of different phenotypes of PCOS (other than the classical type, which has more risk of developing CVD risk), and consideration of varied CVD outcomes [14].

**Figure 2:**
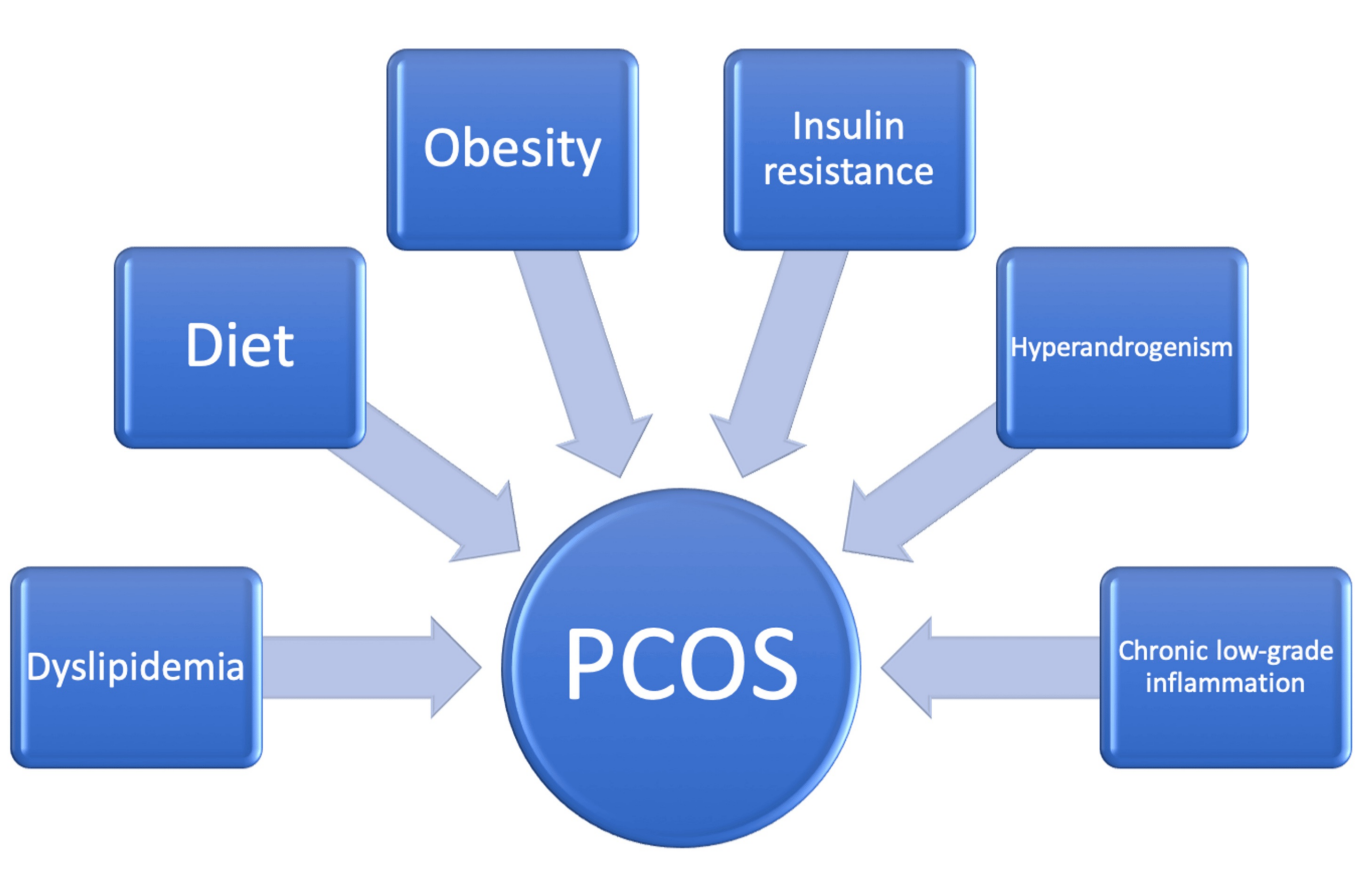
Factors related to enhanced cardiovascular risk in women with polycystic ovary syndrome PCOS: polycystic ovary syndrome

In the following sections, we will discuss the factors commonly associated with the risk of development of CVDs in women with PCOS with relevant supportive data that we found during this systematic review. Factors like obesity, IR, HA, chronic low-grade inflammation, inflammatory markers and mediators, dyslipidemia, the role of genetics, and the role of diet are shown in Figure 2 and discussed below.

Obesity

Overweight and obesity are defined as a body mass index (BMI) within the range of 25.00-39.99 km/m^2^ [21]. Up to 40-80% of women with PCOS are overweight or obese [4,11]. A study shows that obesity is more prevalent in women with PCOS than in women without PCOS [22].

Women dealing with PCOS have an increased accumulation of adipose tissue in the abdominal area, which increases the risk of adverse metabolic syndrome [22]. Obesity plays a central role in the clinical manifestations of PCOS, and therefore, the first-line treatment is aimed at weight loss. Weight reduction is associated with improvements in signs and symptoms seen in this endocrine disorder syndrome [24].

The major fat depots in the affected women with PCOS are characterized as subcutaneous adipose tissue (SAT) located beneath the skin and visceral adipose tissue (VAT), which lines the internal organs. It has been noted that VAT was positively associated with elevated androgen levels and cardiometabolic complications in this demographic [24]. Moreover, in lean women living with PCOS, there is a more significant accumulation of abdominal fat compared with lean controls, which can also result in increased inflammatory markers, which puts them at an increased risk of accelerated atherogenesis [2]. Inflammatory markers were also found to be high in obese patients with PCOS as compared to non-obese patients with PCOS [25].

Insulin Resistance (IR)

IR and consequent hyperinsulinemia are a common finding in PCOS patients, and one of the most common causes of IR is obesity and overweight [9,11]. However, IR within these subjects is also proven to be independent of weight; therefore, lean patients can also display hyperinsulinemia, ultimately leading to T2DM [9,10]. Ninety-five percent (95%) of obese patients with PCOS and 75% of lean women with PCOS have IR [14]. Studies have also found a higher rate of T2DM in this disease population than controls [10].

IR occurs due to glucose metabolism impairment or disturbance and contributes to the risk of evolution of hypertension, dyslipidemia, endothelial dysfunction, atherosclerosis, and vascular smooth muscle hypertrophy, enhancing CVR [9,11,22]. IR is a strong predictor of atherosclerotic CVD [6].

PCOS patients with IR often have systemic inflammation. As a result of IR, reactive oxygen species (ROS) concentration is increased, and associated hyperglycemia results in the activation of inflammatory factors in the body, a finding commonly seen in these patients [4]. An appropriate level of ROS is required in the body for the regulation of transcription factors and antibacterial and anti-inflammatory effects; however, if the level of ROS exceeds, it results in oxidative stress [26], which is discussed later in this article.

The metabolic abnormalities seen in women with PCOS eventually contribute to the development of endothelial dysfunction, atherosclerotic CVD, and atherogenic dyslipidemia [2,7,11,18]. Furthermore, Insulin has a gonadotropin-like action and may increase androgen production, which worsens metabolic syndrome [9].

Hyperandrogenism (HA)

HA in patients with PCOS has a clear association with an increased prevalence of cardiometabolic disorders [9,10]. HA affects up to 75% of women within this demographic [19].

A retrospective study by Falcetta et al. [7] on 602 women with PCOS found that in the younger age group (<20 years old), ovarian dysfunction and HA were commonly seen. Metabolic disturbances were frequently encountered in the older age group (>30 years old). However, they found that metabolic abnormalities were present in all PCOS subjects regardless of age. The clinical and biochemical features of the syndrome also change with aging. The study also states that the androgen levels decrease in women as age progresses; therefore, the signs and symptoms manifested due to HA also decrease or become less evident. There is a contribution of characteristic HA seen in women with PCOS in the development of CVR [7]. In some studies, even younger women in the reproductive age group with excess androgens were found to have endothelial dysfunction [7,19]. In PCOS women, HA potentiates inflammation and increases the risk of atherogenesis [2].

Hyperandrogenemia increases carotid intima-media thickness (CIMT) and the risk of calcification of coronary and aortic arteries due to dyslipidemia-induced HA. High BP is also associated with increased androgen levels and with the progression of atherosclerosis [22].

Chronic Low-Grade Inflammation and the Role of Different Markers

Among women with PCOS, there is a disturbance in oxidant-antioxidant balance [6]. There is an increase in inflammatory mediators like tumor necrosis factor-alpha (TNF-alpha), interleukin-1 (IL-1), and interleukin-6 (IL-6), while superoxide dismutase (SOD) decreases. The increased inflammation is due to HA and obesity. This proinflammatory environment increases CVR, vascular atherogenesis, dyslipidemia, IR, and eventually T2DM [2,4,6,26]. It has also been reported that low-grade inflammation stimulates polycystic ovaries to produce more androgens and contributes to an elevated risk of blood vessel problems [9].

Some inflammatory markers and mediators associated with PCOS are discussed below:

Proinflammatory markers like C-reactive protein (CRP), TNF-alpha, IL-6, plasminogen, endothelin-1, and fibrinogen are increased in patients with IR, which play a vital role in enhancing the risk for increased atherosclerosis and reducing vascular reactivity while impairing endothelial function [4,13,27].

A theory that CRP may be a marker of the intravascular inflammatory process and is one of the most important predictors for the development of CVDs is being researched thoroughly [26]. CRP is increased in obese women with PCOS as compared to normal-weight non-PCOS women, a finding also seen in the adolescent patient age group [25]. Lean women with PCOS also have increased CRP levels, which are milder as compared to PCOS women with obesity. It proves that lean women experiencing this syndrome are also at risk of developing CVDs [2].

Cystatin C is an extracellular cysteine protease inhibitor, a low molecular weight cationic protein; it is a strong predictor of all-cause mortality, including asymptomatic coronary artery disease and advanced metabolic syndrome [6,28]. Ozden Ozdemir et al. [6] studied the relationship between cystatin-C levels, inflammatory, oxidant, and antioxidant markers. Parameters like TNF-alpha, IL-1, IL-1B, IL-6, and malondialdehyde (MDA), which is a risk factor for CVDs, SOD (an antioxidant), and cystatin C, were evaluated by the ELISA method. The authors found that the level of cystatin C in women with PCOS was significantly higher when compared with healthy women.

The disease demographic also had higher inflammatory markers like IL-1B, IL-6, TNF-alpha, and MDA. Meanwhile, SOD was low as compared to controls. Inflammatory markers have also been known to increase androgen secretion and increase the risk of CVDs, which could play a crucial role in the pathophysiology of PCOS [6].

Fetuin B is a peptide hormone that belongs to the cystatin superfamily of cysteine protease [3,29]. It is understood that fetuin B interferes with insulin action and has been linked to IR. Data show that increased levels of fetuin B have been linked to T2DM, atherosclerosis, and risk for inflammation in vascular plaque, causing plaque instability. In a study by Asli et al. [3], 80 women with PCOS and controls were recruited to study fetuin B levels, metabolic-hormonal parameters, and CIMT. The authors found that fetuin-B levels were significantly increased in women with PCOS in general; however, the fetuin-B levels were much higher in PCOS women with co-existing IR as compared to women suffering from PCOS without IR. As per the authors, since CVR is increased in patients living with PCOS and increased levels of fetuin-B are seen in that population, it is fair to consider an association between the two [3]. This evidence is also supported by other literature that has reported similar findings [29,30].

A case-control study by Orkide et al. [1] explored the relationship between PCOS, vascular endothelial growth factor (VEGF), and endostatin, among other inflammatory markers. VEGF is a glycoprotein that is one of the mediators responsible for angiogenesis formation. The literature suggests that angiogenesis is crucial for follicular growth and corpus luteum development, but if angiogenesis fails to regress, polycystic ovaries may develop. The authors found that VEGF was 1.3 times higher in subjects with PCOS as compared to healthy controls, suggesting a role of VEGF in the pathogenesis of polycystic ovaries.

Endostatin has an anti-angiogenic effect on the body and is a cardiovascular marker reflecting vascular and myocardial damage. Authors have found that endostatin levels were increased in PCOS demographics compared to healthy controls. This signifies that elevated endostatin levels correlate to a worsened cardiovascular prognosis in PCOS-affected women [1].

TNF-alpha has been linked with HA, IR, and obesity. In addition, data have shown that serum TNF-alpha increases in PCOS patients [4].

Some metabolites are found to be abnormal in PCOS subjects, which draws attention to the gut microbiota, and one of them is trimethylamine-N-oxide (TMAO) [31]. TMAO is a bacteria or microbiota-derived metabolite that is a biomarker for CVR [13]. The gut microbiota produces TMAO via the metabolism of dietary L-carnitine, choline, betaine, and phosphatidylcholine, which are found in foods like eggs, red meat, and fish [13,31,32]. High levels of TMAO are found in patients with obesity, vitamin D deficiency, and metabolic syndrome and are known to have a pro-atherosclerotic effect [13]. It is positively correlated with an increased risk of atherosclerosis, heart failure, infarct relapse, platelet aggregation, promoting inflammation, and endothelial dysfunction [13,32]. Evidence of altered gut microbiota in women suffering from PCOS resulting in dysbiosis has been discussed in modern literature [13,31,32]. The cause of dysbiosis among these women is thought to be due to excess androgen levels, which could also be independent of diet [31]. An association of increased inflammatory markers with increased levels of TMAO among women with polycystic ovaries in the absence of HA has also been found [32]. A narrative study by Annunziata et al. [13] concludes that TMAO levels are increased in women diagnosed with PCOS and signify a higher possibility of the development of CVR factors in these women. However, further elaborate studies are needed to determine TMAO as a potential biomarker for PCOS [31].

Oxidative stress is high in women dealing with PCOS [4]. Oxidative stress is an imbalance between oxidation and antioxidation that adversely affects cellular metabolism [26]. Oxidative stress also plays a central role in the pathogenesis of PCOS. Amino acid levels of methionine, cystine, isoleucine, phenylalanine, valine, tyrosine, proline, glycine, lysine, and histidine were found to be lower, and arginine and alanine levels were found to be higher in women diagnosed with PCOS which is suggestive of higher metabolic and oxidative stress in this population [27].

Chronic low-grade inflammation may be mediated through adiposity and higher levels of androgens [26]. The relationship between androgens and chronic low-grade inflammation is reciprocal, but it is unclear if high androgen levels in PCOS lead to chronic low-grade inflammation or vice versa [33]. Therefore, chronic low-grade inflammation in patients with PCOS needs strict monitoring to observe endothelial damage, atherosclerosis, and CVDs [33].

Dyslipidemia

Among women affected by PCOS, there are characteristic elevated levels of serum triglycerides and total cholesterol and decreased HDL cholesterol, although this is not a diagnostic criterion [2,11,12,15,18,31].

Lipid-induced inflammation plays a vital role in patients with PCOS, especially when obesity is present. Both inflammation and lipoprotein abnormality can result in accelerated atherogenesis [9,2].

In a case-control study conducted in Iran by Farzaneh et al. [18], 120 subjects with PCOS and 120 healthy controls were matched based on age and BMI. The results showed that patients living with PCOS had markedly higher triglyceride levels (77.5%) and significantly lower HDL-C levels (97.5%) compared to healthy controls [18].

HDL-C particles can decrease cholesterol efflux capacity. It is also anti-atherogenic, anti-inflammatory, antithrombotic, and anti-oxidative [1,6,15]. Reduced HDL-C in PCOS women increases the risk of atherosclerotic CVDs [15]. Investigators mention that central obesity, low HDL-C, and high triglycerides were significant predictors of metabolic syndrome in this population compared to other risk factors [18].

Similar findings were noted in a case-control study by Butler et al. [15]; the authors conducted a proteomic analysis of proteins essential in lipid metabolism for HDL-C in non-obese, non-insulin-resistant PCOS women compared to their matched controls. HDL-C was found to be significantly lower in obese women with PCOS when compared to normal-weight women with the same syndrome. Systemic inflammation impacts HDL particles and causes changes in their composition, thereby impairing their vasculo-protective effects. The causative association between dyslipidemia, PCOS, and IR is not clear, but chronic systemic low-grade inflammation and increased cholesterol in macrophages are seen in PCOS subjects [15].

Role of Genetics

There is evidence of first-degree female relatives being affected with the same syndrome, with an incidence twice as high, establishing a familial pattern of inheritance [17,27]. The genetic loci of PCOS are associated with neuroendocrine, metabolic, and reproductive pathways and also with genetic associations of menopause, metabolic disorders, depression, and male pattern balding [17]. Twin studies and family studies have shown confounding patterns of inheritance; hence, the genetic pattern of inheritance is unclear [17].

Role of Diet in PCOS

Gonzales et al. [2] investigated and found that ingestion of saturated fats can trigger proatherogenic markers like heat shock protein-70 (HSP-70) and activator protein (AP-1) levels in obese patients with PCOS. Ingestion of saturated fats is associated with increased adiposity; the proinflammatory environment in excess adipose tissue can incite premature atherogenesis. In PCOS, inflammation induced by saturated fat ingestion is linked to HA and hyperlipidemia, which are known to increase CVR [2].

As per a review conducted by Dutkowska et al. [11], the diet suitable for PCOS women includes food with a low glycemic index, which has been proven to improve insulin sensitivity and reduce inflammatory markers like CRP and IL-6. A high protein diet of over 20% of daily energy expenditure is recommended for PCOS women. Protein intake reduces body weight and improves lipid profile parameters like LDL-C, total cholesterol, and triglycerides. Omega-3 fatty acids found in oily fish and seafood are also recommended to improve the periodicity of menstrual bleeding [11]. Processed food contains advanced glycation end products (AGEs) that are proinflammatory and should be excluded from the diet [11]. AGEs are also known to be associated with hyperglycemia, DM, renal insufficiency, atherosclerosis, and HA [26].

What Is CIMT and Is It Useful?

CIMT is a minimally invasive ultrasound measurement of the distal wall of the common carotid artery, and it is a good indicator of CVR [3,19,34]. The thickness of the intima-media develops during a long subclinical period of atherosclerosis; therefore, CIMT can be used to assess CVR and detect atherosclerosis [23,34].

Women living with PCOS have a greater CIMT compared to controls, which indicates subclinical atherosclerosis [13,14,21]. HA, which has a direct effect on the vascular system, has also been suggested as a cause of the increase in CIMT [23]. It has been documented that the increased CIMT among these women was independent of BMI, age, and smoking status [23].

In the study by Asli et al. [3], CIMT was measured using high-resolution ultrasound, and the average maximal CIMT from both common carotid arteries was decided as the CIMT value. The study's CIMT value was higher in PCOS subjects than controls.

Coronary artery calcium (CAC), detected via CT scan, can also detect atherosclerosis in asymptomatic populations. The CAC score was higher in a small study based on the PCOS population. However, more studies in larger PCOS-affected populations are needed to find a more substantial and more significant association [14,19].

What Is an Anti-Mullerian Hormone (AMH), and What Is Its Role in PCOS?

Serum AMH is associated with the severity of PCOS, and it is found to be higher in subjects with higher levels of androgens as it can stimulate the granulosa cells to secrete AMH and inhibit follicular development [31,35]. This could be the reason why AMH is two- to four-fold higher in women with PCOS than in healthy women; however, serum AMH levels cannot be used alone to diagnose PCOS [35,36]. Serum AMH levels can assist in the diagnosis of polycystic ovaries when an ultrasound examination of the ovaries is complex due to obesity or in poor echogenic patients [35].

Non-pharmacological Treatment Options

Women with PCOS suffer from reproductive disturbances along with metabolic syndrome and other comorbidities like psychological challenges, including anxiety-depressive disorders. Therefore, the approach to treatment should be multidisciplinary [11,17]. Considering the adverse health effects of obesity on women with PCOS, weight loss or weight reduction is considered to be the principal line of treatment [11].

The recommendations for the prevention of CVD include modification of lifestyle via exercise, diet, weight control, smoking cessation, and management of BP, blood cholesterol, and blood sugar levels [14].

Based on available data, interventions that can reduce 10-20% body weight are required to prevent conditions like cardiovascular events, prediabetes, DM, and other obesity-related diseases [12]. In obese patients, physical exercise for around 150 minutes, five times a week, with 50-70% of maximum heart rate (HR max = 220-age) is recommended. In non-obese patients, exercise for 15-20 minutes, a minimum of three times a week, and HR max of 60-80% is recommended [11].

In a study by Jafari et al. [4], moderate exercise sessions were conducted for 12 weeks. As a result of the intervention, inflammatory markers in subjects with PCOS, their body weight, BMI, and the risk of IR were reduced, reducing adverse cardiovascular effects.

Vitamin D deficiency is often found in the general population but is more prevalent in PCOS patients; therefore, supplementation of vitamin D is recommended [11,37]. Studies have proven vitamin D's anti-inflammatory effects and its role in increasing insulin synthesis and release; however, there were no significant changes in metabolic, inflammatory, or endocrine parameters after vitamin D supplementations [37,38].

Pharmacotherapy for Patients With PCOS

For weight management, glucagon-like peptide-1 (GLP-1) receptor agonists like liraglutide were approved in 2014 for overweight patients without DM, and in 2021, semaglutide was approved provided they satisfied the criteria of being overweight (BMI above 27 kg/m^2^) and obese (BMI above 30 kg/m^2^) with any adipose-based chronic diseases (ABCD) like hyperlipidemia, dysglycemia, non-alcoholic fatty liver disease, acanthosis nigricans, and unfavorable CVR profile [12].

Combined oral contraceptives (COCs) are the drug of choice to correct androgen-related symptoms and menstrual abnormalities in women with PCOS who are not planning pregnancy [9,39]. However, the use of COCs can be seen as an added CVR factor for women as it has been associated with new-onset hyperglycemia, hypertension, hypertriglyceridemia, elevated CRP levels, and increased chances for venous thromboembolism [9,21,39].

Newer COCs contain ethinyl estradiol (EE), which are low-dose estrogens and progestins [39,40]. Four generations of progestins are available as per their time of development, i.e., first generation (norethindrone family); second generation (norgestrel and levonorgestrel); third generation (desogestrel and gestodene); and fourth generation, which are newer progestins (trimegestone, nestorone, nomegestrol, cyproterone acetate (CPA), and chlormadinone), while drospirenone is derived from spironolactone and dienogest of testosterone [39].

The progestin component of COCs suppresses the luteinizing hormone (LH) secretion by inhibiting ovarian androgen production, and the estradiol component reduces free androgen levels by increasing sex hormone binding globulin (SHBG). COCs also reduce adrenal androgen secretion. This anti-androgenic effect of COCs may improve the metabolic syndrome seen in patients with PCOS, as they may improve the deranged lipid profile [39].

The side effects of COCs include weight gain, increase in BP, venous thromboembolism, stroke, myocardial infarction, abnormalities in glucose metabolism, and mood alterations, among other side effects. COCs are contraindicated in patients with a medical history of hypertension, liver disease, migraine, history of deep vein thrombosis, smokers, and many more [39]. Therefore, the use of COCs should be personalized as per the patient's medical history, CVR profile, menstrual history, and family history [9,39,40].

It has also been noted that adding metformin or myoinositol to COCs can improve insulin sensitivity, fasting glucose levels, BMI, and HA in women with PCOS compared to the individual use of COCs [9,21].

Metformin is an antidiabetic medication that increases insulin sensitivity and peripheral glucose uptake, decreases hepatic gluconeogenesis, and reduces glucose reabsorption. It helps improve lipid profiles and inflammatory markers commonly seen in women suffering from PCOS. Metformin can also be used to improve menstrual irregularity as an alternative to COCs in PCOS patients where COCs are contraindicated [9].

Inositols (myoinositol) are polyols with insulin-sensitizing properties [21]. Myo-inositol is a dietary supplement known to improve HA, IR, and assisted reproduction results [11]. It has also shown improvement in menstrual irregularity, ovulation, and metabolic and oxidative imbalances [5].

Elevated androgen production is seen in PCOS; therefore, depending on the severity of the symptoms, androgen receptor antagonists, 5-alpha-reductase inhibitors, and synthetic progestogens are used to reduce HA [9].

Antiandrogen agents like spironolactone can improve lipid profile and reverse endothelial dysfunction [9]. Ketoconazole, an antifungal agent, has shown improvement in acne and hirsutism and a favorable effect on lipid profile. However, the drug-to-drug interaction risk of this agent must be considered, so it should be used with caution [9]. Finasteride, a 5-alpha-reductase inhibitor, is also helpful in acne and hirsutism [9].

Statins are widely used drugs for treating dyslipidemia [31]. In particular, atorvastatin improves lipid profile and inflammatory reactions and lowers testosterone levels [9,31]. Although the assessment of lipid profile is recommended in women diagnosed with PCOS, more elaborate studies concentrating on the recommendations for the use of statins, specifically in the PCOS demographic, are needed [9,41].

Need for Screening

In the 2023 international guideline for management of PCOS, the recommendation for risk assessment of CVDs was suggested to be included as women with PCOS are at an increased risk for CVD [8]. Given the available data, the increase in cardiometabolic diseases in women with PCOS is evident, which warrants screening for CVR to prevent severe consequences. However, screening in a low-risk population may lead to overdiagnosis and a low yield of preventable cases [10].

Limitations

This systematic review has some limitations, which were noticed by both the authors: The heterogeneity of the diagnostic criteria of PCOS across various literature differed, which may have resulted in overdiagnosis. The search was only conducted on Google Scholar and PubMed; other scientific search engines, such as Cochrane or Science Direct, were not included. Many interesting publications relating to the topic could not be included due to limited accessibility, as we only included free full-text publications. This article did not discuss inflammatory markers, hormonal imbalance, and USG findings in detail, as we wanted to limit the focus to factors affecting CVR in women with PCOS only.

## Conclusions

The etiology and pathophysiology of PCOS are unknown. However, several metabolic disorders are commonly associated with it. We studied that these metabolic disorders enhance the CVD risk in women with PCOS. This is proven by evaluating the levels of various inflammatory markers and metabolites linked to augmented cardiac risk. In this systematic study, we found that in addition to a deranged lipid profile and elevated inflammatory markers like IL-1, IL-6, TNF-alpha, and oxidative stress, CIMT is also high in women suffering from PCOS. Microbiota-derived metabolite like TMAO, which is associated with an increased risk of atherosclerosis, heart failure, and endothelial dysfunction, is raised in these women. Endostatin, which reflects vascular and myocardial damage, is higher in this population. Fetuin B, a peptide hormone linked with atherosclerosis and vascular plaque instability, is also increased in this demographic. After analyzing these parameters, it is safe to conclude that the risk of CVD is higher in women with PCOS as compared to healthy women. We also found that the role of diet in women with PCOS is also crucial in increasing the risk for CVDs.

The need for screening is evident; however, screening in low-risk populations may result in overdiagnosis and a low yield of preventable cases. As per the literature, in young women with PCOS, there is increased CIMT signifying subclinical atherosclerosis, and in premenopausal or menopausal women, androgens decrease, which reduces the signs and symptoms. Therefore, a well-planned large-scale cohort study is recommended to identify the patients who can benefit from the screening interventions. Drugs, like combined oral contraceptives (COCs), are used in women with PCOS who do not desire pregnancy; metformin and myo-inositol are also commonly used to treat insulin resistance and improve fertility. To treat dyslipidemia, statins are widely used; however, the exact recommendation for its use in women with PCOS should be studied.
